# Editorial

**Published:** 1913-05

**Authors:** 


### EDITORIAL

The Eusiness Office of the *Journal-Record* is 23 West Alabama Street.

The Editorial Office is 1014-15 Atlanta National Bank Building.

Address all Business Communications to *Journal-Record of Medicine*, 23 West Alabama Street.

Make remittances payable to *The Journal-Record of Medicine*.

On matters pertaining to the Editorial and Original Communications, address Edgar G. Ballenger, M. D., Atlanta, Ga.

Reprints of Original Articles will be furnished at cost price. Requests for the same should always be made in THE MANUSCRIPT.

We will present, postpaid, on request to each contributor of an original article, twenty (20) marked copies of *The Journal-Record of Medicine* containing such article.

### GREAT PROSPECTS.

Modelled after America's most successful surgical and clinical association, the Georgia Surgeons Club will hold clinics in Atlanta Oct. 15 and 16, 1913. The Georgia Surgeons Club is a new organization with great prospects; it has been launched with enthusiasm and backing sufficient to make it a great success. The plan is similar to that so successfully followed by the American Congress of Surgeons. Physicians and surgeons who wish to see work, modern, up-to-date, practical, scientific work, actually done can see it if they come to Atlanta on the above dates.

The notable success of the American Congress of Surgeons means that similar miniature societies will be organized throughout the various states. Let Georgia be one of the leaders in this important movement, which is not to supplant existing associations but to supplement them.

The following advisory committee has been appointed to arrange clinics: Dr. W. S. Elkin, Dr. W. P. Nicholson, Dr. F. W. McRae, Dr. W. A. Crowe, Dr. J. N. Ellis, Dr. Geo. H. Noble, Dr. J. G. Earnest, Dr. W. C. Jarnigan, Dr. W. F. Westmoreland, Dr. W. S. Goldsmith, and Dr. E. C. Davis. A surgeon of note has been invited to address the club at the evening session. Further details will be published from time to time. Begin now to arrange your plans to attend this meeting.

### THE PRESIDENT'S AND SOUTHERN MEDICAL ASSOCIATION SPECIAL.

Physicians in and around Atlanta who intend to attend the meeting of the American Medical Association in Minneapolis, June 17-20, 1913, are requested to send their names to Dr. Seale Harris, Mobile, Alabama. Most attractive plans are being arranged so that we will have the Presidents Special from Nashville combined with the Southern Medical Association Special. By this means a very pleasant trip can be made in special charge of passenger representatives of the roads in charge. If we secure enough passengers, the Atlanta Sleeper will be attached to the special train in Nashville, the arrangements allow one day in Chicago.

To Members American Medical Association.

That my friends desiring to attend the meeting of the American Medical Association in Minneapolis might have every comfort, I have taken the responsibility of arranging for them a special train to be run from Nashville to Minneapolis, allowing a stop-over of one day in Chicago, thereby breaking the trip and allowing the party an opportunity of seeing Chicago.

The Louisville & Nashville, Chicago and Eastern Illinois, and Chicago, Milwaukee and St. Paul Railroads have promised to arrange a train de luxe in every feature which will be designated "The President's Special." This train will leave Nashville on June 15th, at 7:50 p.m. on June 16th and arriving at Minneapolis at 7:45 a.m. in ample time for breakfast before the opening session

I am very much interested in seeing a large attendance at our Minneapolis meeting, and in order that we may have the comfor of the best available trains and schedule, together with the pleasure of a train exclusively ours, I would urge that you make your reservations as soon as possible.

Commencing June 1st, there wil lbe round trip summer touritss' rates from all points to Minneapolis, and this will be the best ticket for you to buy. Purchase your ticket at your home station, and see that it reads via Louisville and Nashville,

Chicago and Eastern Illinois, and Chicago, Milwaukee and St. Paul.

We have assurances that this train will be in special charge of the pasesnger representatives of the above roads, and your pleasure and comfort en route will be their first consideration.

For those who cannot join the party in Nashville, arrangements can be made to join this SPECIAL in Chicago.

For all reservations in this "President's Special" and full information concerning the trip, write to Perry Bromberg, M.D., Secretary Tennessee State Medical Association, Nashville, Tenn. and he will be glad to make reservations for you.

Respectfully,
J. A. WITHERSPOON, Pres. A. M, A,

---

Attention is once more called to the unique plan whereby a great organization of medical men pays another great compliment of a visit en masse. Arrangements have been made fo rspecial trains of Pullman sleepers starting from Southwestern, Southern and Southeastern points to assemble at Louisville bearing members of the Soutehrn Medical Association and their friends.

The trains will be elegant and sumptuous and will form such a procession as the most luxurious travel has ever seen. Efforts will be made to procure stop-overs for entire trains at Mammoth Cave long enough to allow visitors to inspect that wonder of the world, and at all the cities of any size through which they pass, rest periods will be enjoyed.

The excursion promises to be one of the most successful of its kind ever undertaken. Already, physicians from various states are communicating with the Secretary of the Southern Medical Association, Dr. Seale Harris, of Mobile, concerning rates and regulations, and others who contemplate taking the trip should lose no time in notifying him of their intentions and securing reservations.

The Louisville & Nashville Railroad has undertaken tot provide all the transportation required, but the affair promises to be so extensive that it will be necessary for them to know well in advance the approximate number for which they must provide cars. It is proposed that these shall be of the latest and most luxurious character, yet the rates will be temptingly moderate. Southern doctors who miss this opportunity to enjoy such a combination of travel, pleasant association, wide observation and profitable discussion of professional subjects will have something to regret for a long time.

Doctor, as soon as you read this write to Dr. Harris and take steps to secure accomodations for yourself and your better half.

